# Melatonin’s Effect in Febrile Seizures and Epilepsy

**Published:** 2014

**Authors:** Abolfazl MAHYAR, Parviz AYAZI, Reza DALIRANI, Nargess GHOLAMI, Mohammad Mahdi DANESHIKOHAN, Navid MOHAMMADI, Mohammad Hossein AHMADI, Ahmad Ali SAHMANI

**Affiliations:** 1 Department of Pediatrics, Qazvin University of Medical Sciences, Qazvin, Iran.; 2 Department of Pediatric Nephrology, Mofid Children’s Hospital, Shahid Beheshti University of Medical Sciences, Tehran, Iran.; 3 Department of Community Medicine, Tehran University of Medical Sciences, Tehran, Iran.

**Keywords:** Melatonin, Simple febrile seizures, Complex febrile seizures, Epilepsy

## Abstract

**Objective:**

Recognition of risk factors for febrile seizures (FS) and epilepsy is essential.

Studies regarding the role of melatonin in these convulsive disorders are limited.

This study determines the relationship between serum melatonin levels and FS and epilepsy in children.

**Materials & Methods:**

A population of 111 children with simple FS, complex FS, and epilepsy (37 children per group, respectively) were included as case groups. In addition, 37 febrile children without seizures comprised the control group. Serum melatonin levels were measured and compared between all groups.

**Results:**

The serum melatonin levels in the simple, complex FSs, and epilepsy groups were 2, 2.4, and 2 pg/ml, respectively. The serum melatonin level in the control group was 2.1pg/ml.

Moreover, there were no significant differences observed while comparing the case groups.

**Conclusion:**

The present study reveals that there is no association between serum melatonin level and simple or complex FS and epilepsy. It appears that melatonin plays no significant role in these convulsive disorders.

## Introduction

Febrile seizures (FS) and epilepsy are the most common causes of seizures in children (1).The incidence of FS is2−9% (2,3).According to the definitions provided by the National Institutes of Health (NIH) in 1980 and the International League Against Epilepsy (ILAE) in 1993,FS refers to a seizure that occurs following an increase in body temperature(typically over 38°C). In addition, these patients have no central system infection or electrolyte imbalance (2–5). Epilepsy is considered present when 2 or more unprovoked seizures occur at an interval greater than 24h apart. The cumulative lifetime incidence of epilepsy is 3% with more than half of cases beginning in childhood. Epileptic children have no fever or central nervous system infection (6–9).Despite numerous studies, the actual causes of these convulsive disorders remain unknown (10, 11).Interestingly, Guo and Yao reported that serum melatonin levels decreased significantly in children with complex FS and epilepsy(12).Melatonin is a tryptophan-derived hormone that is primarily secreted from the pineal gland (13,14).The present study investigates the relationship between serum melatonin levels and simple or complex FS and epilepsy in children.

## Materials & Methods

This case-controlled study was conducted at Qazvin Children’s Hospital, affiliated with Qazvin University of Medical Sciences (Iran) in 2010. Qazvin Children’s Hospital is the only referral hospital for children in Qazvin province. Case groups (111 total patients) were selected consecutively among children who were admitted to the hospital following simple or complex FS and epileptic seizures (37 patients per group, respectively). The control group was comprised of 37 febrile children without seizures. The age of all patients was 6-months–5-years of age. The Sample size was calculated according to the following: α=0.05; β=0.01; μ2=23.93 ng/1μ 1=20.72 ng/1; δ1=2.54; δ2=2.01 (12). Inclusion criteria for the FS groups were as follows: 1) fever ≥38°C; 2) the occurrence of seizures meeting the criteria for simple FS(generalized seizure and s lasting less than15 min);and 3)the occurrence of seizure meeting the complex FS criteria (focal, lasting more than15 min, and repeated more than once within 24h).Epilepsy was considered to be present when 2 or more unprovoked seizures occurred at an interval greater than 24 hapart (16,7).Patients with central nervous system infections (such as meningitis or encephalitis), electrolyte imbalances, or neurological deficits were excluded. The control group included healthy children without seizures who visited the hospital clinic due to mild febrile illness without any intervention. Children in all groups were matched in terms of age, gender, weight, height, head circumference, and fever severity. Weight, height, head circumference, and body temperature (axillary) were measured according to standard methods (6).All patients lived in Qazvin City and were permanent

residents. The study was approved by the ethical committee of the Research Department in the Qazvin

University of Medical Sciences (Project No.232).

All parents were provided information regarding the research method in simple language. The children were included in the study after their parents agreed and signed the informed consent form. In all groups, 6 mL of blood was drawn from the peripheral vessels and centrifuged. 

The serum was then poured into an acid-washed tube and kept in the refrigerator at a controlled temperature (-20°C). All melatonin samples were collected within 24 h after clinical seizures. All conditions such as postural conditions and environmental lighting were the same for all groups during blood sampling (15).

Measurement of serum melatonin was performed by enzyme-linked immunosorbent assay (ELISA) with a kit (IBL International, Hamburg, Germany, and LotNo. 

EME151). To improve accuracy, all samples were measured in duplicate.For statistical analysis, analysis of variance (ANOVA) was used to compare variables between the case and control groups; the Mann-Whitney test was used for comparisons of serum melatonin levels. 

SPSS version11.5 was used for data analysis. A P-value of <0.05was considered statistically significant.

**Ethics**

The ethics committee of the research department in the Qazvin University of Medical Sciences (Project No. 232) approved the study. All parents were provided information regarding the research method in simple language. The children were included in the study after their parents agreed and signed the informed consent form.

## Results

The simple FS group was comprised of 19male patients and 18 female patients. The complex FS and epilepsy groups were comprised of15 males and 22 females, and 21 males and 16 females, respectively. In the control group, 16patients were male and 21 were female (p = 0.43). The minimum and maximum ages in the case and control groups were 6- and 60-months old, respectively. 

There were no statistically significant differences between the groups in terms of age, weight, height, head circumference, and body temperature (p>0.05; Table 1). The serum melatonin levels of the simple and complex FS and epilepsy groups were 2, 2.4, and 2 pg/ml, respectively. The serum melatonin level of the control group was 2.1pg/ml. There were no significant differences in serum melatonin levels between patients with simple FS (p=0.433), complex FS (p= 0.485), and epilepsy (p=0.192) relative to the control individuals. 

**Fig1:**
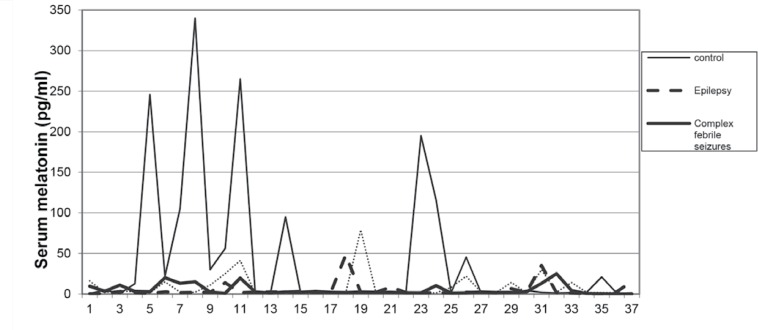
Comparison of serum melatonin level in case and control groups

Furthermore, there were no significant differences observed while comparing the case groups (p>0.05;Table2, Figure1).

## Discussion

Although several studies regarding the role of the numerous risk factors for FS and epilepsy have been reported (10,11,16), the actual causes of these convulsive disorders remain unknown. Based on the role of some antioxidants in FS (11–16), present study determines whether another antioxidant, melatonin, is involved in FS and afebrile seizures. As far as our knowledge and the literature review are concerned, studies in this area are limited (11,17,19).Melatonin ((N-acetyl-5-methoxytryptamine) was first extracted from the bovine pineal gland by Aaron Lerner in 1958. 

**Table1:** Comparison of Variables in Case and Control Groups

**Variables**	**Case groups**			**Control group** **(Mean ±SD)**	**P-Value**
	**Simple febrile seizures** **(Mean ±SD)**	**Complex febrile seizures** **(Mean ±SD)**	**Epilepsy** **(Mean ±SD)**		
**Age(month)**	23.75 ±14.59	31.41±1602	28.21 ± 15.17	24.20±14.46	0.1
**Weight(kg)**	12.57±2.46	13.22±2.26	13.40±2.40	12.14±2.14	0.09
**Height(cm)**	84.63±10.05	89.58 ±11.34	87.91±11.35	84.44±10.78	0.07
**Head circumference(cm)**	47.68± 1.93	48.54±2.07	48.22±1.97	47.66 ± 1.83	0.1
**Temprature** ^0^ **c**	38.40± 0.36	38.42± 0.42		38.25± 0.39	0.1

**Table2:** Comparison of Serum Melatonin between Groups

**Groups Serum melatonin (median** ** p** **g/ml)**	**Groups Serum melatonin (median pg/ml)**	**P Value**
Control (2.1)	Simple FS (2)	0.43
	Complex FS(2.4)	0.48
	Epilepsy (2)	0.19
Simple FS (2)	Complex FS (2.4)	0.92
	Epilepsy (2)	0.4
Complex FS (2.4)	Epilepsy (2)	0.24

Melatonin is a major hormone secreted by the pineal gland that is synthesized from tryptophan and serotonin (20).Although the principal function of melatonin is the regulation of circadian rhythms and seasonal responses (20). Several studies have described its antioxidant properties (21–24).The reported antioxidant activities of melatonin include scavenging reactive oxygen specimens such as hydroxyl radicals and nitric oxide, inhibition of lipid peroxidation, decreased oxidation products such as malonealdehyde, increased antioxidative defense system enzymes such as copper and zinc superoxide dismutases, glutathione peroxidase, and glutathione reductase, increased intracellular glutathione levels, and inhibition of the peroxidative enzymes such as nitric oxide synthase. The authors of these studies regarding these mechanisms suggest that the function of melatonin’s antioxidant activity is to maintain the health of the central nervous system (17,21–24). However, the notion of anticonvulsant properties of melatonin are controversial. Guo etal. reported that serum melatonin levels were decreased in children with epilepsy or complex FS. They advised that administration of exogenous melatonin might be useful for the treatment of epilepsy and FS in children (12).A study reported by Bazil etal. in patients with intractable temporal lobe epilepsy revealed that salivary melatonin was reduced in patients with epilepsy at a baseline relative to controls, which increased 3-fold following seizures. This study indicated that melatonin has anticonvulsant properties (17). Another study showed that melatonin levels were low in patients with nocturnal and diurnal complex partial epilepsy relative to controls (18).A separate study conducted by Molina-Carballo etal. in 54 children with convulsive crisis (febrile and epileptic) showed that serum melatonin levels increased during seizure attacks and returned to normal values 1 hlater. They further concluded that excitation of melatonin generation by a convulsive crisis may represent the body’s response to seizures and is aimed at achieving homeostasis (25).Similar results were found in another pervious study (26). However, a study reported by Schapel et al. in 30 patients with untreated active epilepsy and 19healthy controls showed that the excretion rates of urinary 6-sulfatoxymelatonin (a hepatic metabolite of melatonin) in patients with active epilepsy were greater than for healthy controls. They further concluded that melatonin generation is increased in untreated patients with active epilepsy and has a circadian model with phase differences relative to controls (19).In contrast, Rao et al reported that during seizures and 2 h afterwards, serum melatonin levels did not change and remained within the normal limits of healthy populations (27). Furthermore, a separate study demonstrated that there were no significant differences in salivary melatonin levels between FS and epileptic patients relative to control individuals. They also concluded that the anticonvulsant effect of melatonin in epilepsy and FS is not significant (15).However, Fauteck showed that single evening dose of 5−10 mg melatonin could reduce the occurrence of epileptic attacks in children. This study further suggested that melatonin could be a useful antiepileptic drug (28).

The antiepileptic effect of melatonin was confirmed by Peled, who indicated that the anticonvulsant proprieties of melatonin are due to antioxidant activity, increases of brain gamma-aminobutyric acid (GABA) concentration, inhibition of calcium influx into neurons, and decreased neuronal nitric oxide generation(29).In contrast, another study found that the anticonvulsant effect of melatonin was negligible, and may sometimes exacerbate seizures (30).The present study revealed that there are no associations between serum melatonin levels and simple or complex FS and epilepsy. It appears that melatonin plays no role in these convulsive disorders. 
